# FICC-Seq: a method for enzyme-specified profiling of methyl-5-uridine in cellular RNA

**DOI:** 10.1093/nar/gkz658

**Published:** 2019-07-30

**Authors:** Jean-Michel Carter, Warren Emmett, Igor Rdl Mozos, Annika Kotter, Mark Helm, Jernej Ule, Shobbir Hussain

**Affiliations:** 1 Department of Biology and Biochemistry, University of Bath, Claverton Down, Bath, BA2 7AY, UK; 2 The Francis-Crick Institute, 1 Midland Road, London, NW1 1AT, UK; 3 University College London Genetics Institute, Gower Street, London, WC1E 6BT, UK; 4 Department for Neuromuscular Diseases, UCL Queen Square Institute of Neurology, London WC1N 3BG, UK; 5 Johannes Gutenberg-Universität, Institut für Pharmazie und Biochemie, Staudinger Weg 5, 55128 Mainz, Germany

## Abstract

Methyl-5-uridine (m5U) is one the most abundant non-canonical bases present in cellular RNA, and in yeast is found at position U54 of tRNAs where modification is catalysed by the methyltransferase Trm2. Although the mammalian enzymes that catalyse m5U formation are yet to be identified via experimental evidence, based on sequence homology to Trm2, two candidates currently exist, TRMT2A and TRMT2B. Here we developed a genome-wide single-nucleotide resolution mapping method, Fluorouracil-Induced-Catalytic-Crosslinking-Sequencing (FICC-Seq), in order to identify the relevant enzymatic targets. We demonstrate that TRMT2A is responsible for the majority of m5U present in human RNA, and that it commonly targets U54 of cytosolic tRNAs. By comparison to current methods, we show that FICC-Seq is a particularly robust method for accurate and reliable detection of relevant enzymatic target sites. Our associated finding of extensive irreversible TRMT2A-tRNA crosslinking *in vivo* following 5-Fluorouracil exposure is also intriguing, as it suggests a tangible mechanism for a previously suspected RNA-dependent route of Fluorouracil-mediated cytotoxicity.

## INTRODUCTION

High resolution genome- and transcriptome-wide mapping of RNA modifications has made many advances and received considerable attention in the molecular genetics field in recent years. Despite this, one of the most pressing questions remains adequate characterization of one of the more abundant RNA modification types, methyl-5-uridine (m5U), and in particular, identification of the mammalian enzyme(s) that catalyse its formation (1). Indeed, any functional characterization of mammalian m5U currently remains extremely limited in the literature, though relevant efforts would likely be aided via identification of the genetic factors directly responsible for catalysing its formation. In bacteria and yeast, the enzymes TrmA and Trm2, respectively, are known to target U54 of tRNAs for methylation (2,3). Based on sequence homology to TrmA and Trm2, two mammalian m5U-catalysing enzymes are predicted, TRMT2A and TRMT2B. However, whether these enzymes represent true m5U methyltransferases, and indeed what their respective site-specificities are, remains to be demonstrated.

Only two of the current high throughput sequencing-based basic methodological principles used to map RNA modifications are able to identify sites inherently in an RNA modification enzyme-specified manner (4). Both initially described in 2013, these comprise the Aza-IP method used to identify NSun2- and Dnmt2-dependent human methyl-5-cytosine (m5C) sites (5), and the methylation-iCLIP (miCLIP)-based approach previously used to map NSun2-dependent human m5C (6) and more recently RlmN-dependent bacterial methyl-2-adenosine (m2A) sites (7). Such methods are centred on ‘catalytic crosslinking’ principles, where the aim is to stabilize and capture catalytic intermediates of the *in vivo* methylation reaction (8). This is achievable, as during the normal catalytic reaction scheme, the nucleotide undergoing methylation becomes covalently crosslinked to the enzyme. In Aza-IP the use of the cytosine analogue 5-azacytidine is used to stabilize the crosslinked intermediates, whereas in miCLIP, use of a specific point mutant form of the enzyme enables crosslink stabilization (9).

**Figure 1.**
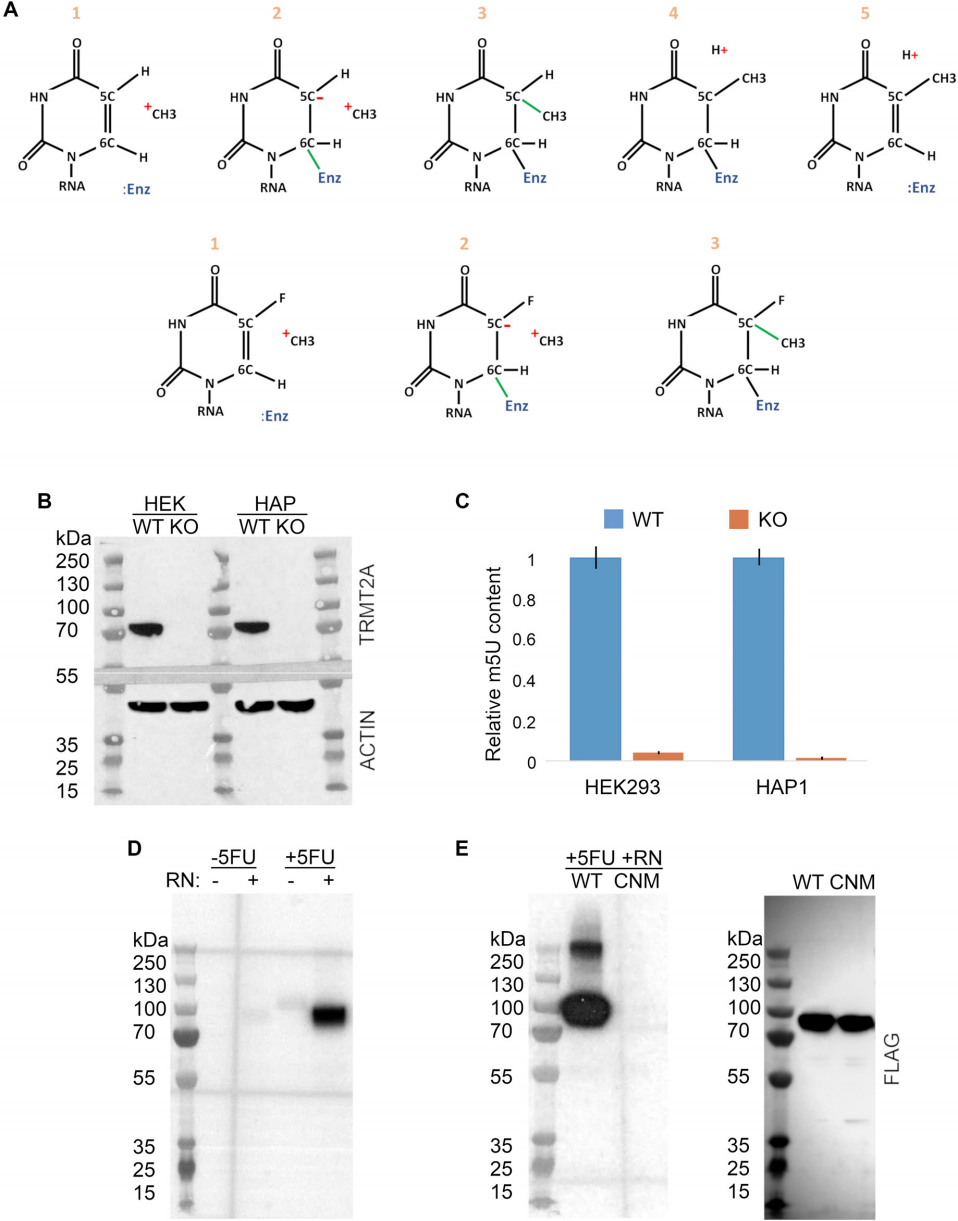
TRMT2A is an m5U methylase, and 5-Flurorouracil can be used to trap its enzymatic target RNAs. (**A**) The ‘catalytic crosslinking’ principle for the m5U modification using 5FU is depicted. During modification of normal uridine residues (top), an m5U methyltransferase (:Enz) becomes covalently crosslinked to the carbon at position 6 of the base ring. This renders the carbon at position 5 susceptible to electrophilic attack, and a methyl group (supplied by an S-Adenosyl-Methionine donor, not shown) is transferred to it. Proton abstraction from the 5-carbon occurs next (in an enzyme-dependent manner) before β-elimination of the m5U methyltransferase from the 6-carbon. During modification of 5FU residues (bottom), enzyme-RNA crosslinking and methyl transfer steps occur normally, but the enzyme cannot mediate fluoride abstraction from the 5-carbon and hence its release from the 6-carbon does not occur either. (**B**) Anti-TRMT2A Western of lysates prepared from HEK293 and HAP1 TRMT2A knockout cell lines and control parent cells (top); anti-actin loading control from the same blot is also shown (bottom). Predicted molecular weight of the native TRMT2A protein is 69 kDa, whilst that of actin is 42 kDa. (**C**) Mass spectrometry-based relative quantifications of m5U presence in total RNA from parent (WT) versus TRMT2A knockout (KO) cells. Experiments were performed in triplicate, and for each LC-MS measurement, 1 μg of total RNA sample and 100 ng *Escherichia coli* internal standard was used. (**D**) HEK293 cells were treated with 100 μM 5FU for 24 h or mock-treated, and immunoprecipitations next performed using anti-TRMT2A antibody. All samples were treated with DNase, and some samples as indicated were also treated with RNase (RN). RNA components of purified complexes were radioactively labelled, and complexes then run on a polyacrylamide gel. In samples not treated with RNase, protein–RNA complexes do not efficiently undergo electrophoresis, and are thus not readily observable. However, in RNase-treated samples, where the RNA component of complexes is digested to a minimal size, clear bands at just above the predicted molecular weight of TRMT2A are observed following 5FU treatment. (**E**) FLAG-tagged TRMT2A-WT (WT) or TRMT2A-catalytic nucleophile mutant (CNM) (see Supplementary Figure S2) were ectopically expressed in HEK293 cells, and an anti-FLAG antibody was used for the immunoprecipitations. Use of the CNM abolishes the observation of 5FU-induced TRMT2A-RNA crosslinked intermediates (autoradiograph, left). Control anti-FLAG Western analysis confirmed that TRMT2A-CNM was expressed at similar levels to TRMT2A-WT (blot, right).

Here we identify TRMT2A target sites in a genome-wide single-base resolution manner through catalytic crosslinking principles to develop a technique termed ‘Fluorouracil-Induced-Catalytic-Crosslinking-Sequencing’ (FICC-Seq). The method works by incubating cell cultures with 5-Fluorouracil (5FU); fluorouridine triphosphate (FUTP), an active metabolite of 5FU, then becomes incorporated into nascent RNA in competition with uridines during gene transcription (10,11). Thus when an m5U methyltransferase proceeds to modify such residues it becomes covalently crosslinked to them as a normal step of the usual catalytic reaction (Figure 1A) (12–14). Although FUTP residues are successfully methylated (15), normal substrate release does not occur, resulting in a permanent enzyme-RNA catalytic intermediate stabilized via a covalent crosslink occurring on the modified base (Figure 1A) (15–17). Reverse transcription of such RNAs will very efficiently stall at the peptide-RNA crosslink sites, thus enabling reliable nucleotide-resolution detection of enzymatic target sites (18). In order to determine the robustness of the FICC-Seq method, we also performed relevant iCLIP (19) and miCLIP experiments in parallel. We thereby characterized TRMT2A as the enzyme commonly responsible for human cytosolic tRNA-m5U54 formation.

## MATERIALS AND METHODS

### Cell lines and cell culture

The HEK293 TRMT2A knockout cell line was generated by GenScript (Hong Kong), and the HAP1 TRMT2A knockout cell line was purchased from Horizon Discovery; knockout status at protein level was confirmed in our laboratory using Western blotting. HEK293 cells were cultured in Dulbecco's Modified Eagles Medium (DMEM) supplemented with 10% Fetal Bovine Serum (FBS) and penicillin/streptomycin. HAP1 cells were grown in Iscove's Modified Dulbecco's Medium (IMDM) supplemented with 10% FBS and penicillin/streptomycin. All cultures were maintained at a temperature of 37°C in a humidified incubator with 5% CO_2_. When required, cells were harvested by washing in phosphate-buffered saline (PBS) and then incubating with Trypsin-ethylenediaminetetraacetic acid followed by further washing of detached cells in PBS.

### Cellular viability assay

Cell viability was assayed using the RealTime-Glo MT cell viability assay kit (Promega). Cell monolayers were seeded at equal densities (∼4000 cells/well) on a 96-well plate and incubated in 40 μl growth media at 37°C for 24 h. The commercial substrate + enzyme mix and the appropriate 5FU dose were combined in 40 μl media before being applied to each monolayer (80 μl total per well) for a final concentration of either 0, 10, 100, 1000 or 10000 μM of 5FU. After an initial incubation of 1 h, the first measurement was taken to establish the baseline and subsequent measurements were taken over a 48 h incubation period using a Pherastar FS microplate reader (BMG Labtech).

### Western blotting

Western blotting was performed as described previously (6). Primary antibodies used were mouse monoclonal anti-TRMT2A (Origene, clone OTI1C3, catalog# TA505555); mouse monoclonal anti-actin (Santa Cruz); mouse monoclonal anti-FLAG (Origene).

### Mass spectrometry

Total RNA samples were digested into nucleotides using 0.6 U nuclease P1 from *Penicillium citrinum* (Sigma-Aldrich), 0.2 U snake venom phosphodiesterase from *Crotalus adamanteus* (Worthington), 2 U FastAP (Thermo Scientific), 10 U benzonase (Sigma-Aldrich), 200 ng Pentostatin (Sigma-Aldrich) and 500 ng Tetrahydrouridine (Merck-Millipore) in 25 mM ammonium acetate (pH 7.5; Sigma-Aldrich) over night at 37°C. The nucleosides were then spiked with internal standard (^13^C stable isotope-labelled nucleosides from *Escherichia coli*, SIL-IS) and subjected to analysis. Triplicates with 1 μg digested RNA and 100 ng internal standard were analysed via LC-MS (Agilent 1260 series and Agilent 6460 Triple Quadrupole mass spectrometer equipped with an electrospray ion source (ESI)). The solvents consisted of 5 mM ammonium acetate buffer (pH 5.3; solvent A) and LC-MS grade acetonitrile (solvent B; Honeywell). The elution started with 100% solvent A with a flow rate of 0.35 ml/min, followed by a linear gradient to 8% solvent B at 10 min and 40% solvent B after 20 min. Initial conditions were regenerated with 100% solvent A for 10 min. The column used was a Synergi Fusion (4 μM particle size, 80 Å pore size, 250 × 2.0 mm; Phenomenex). The UV signal at 254 nm was recorded via a diode array detector (DAD) to monitor the main nucleosides. ESI parameters were as follows: gas temperature 350°C, gas flow 8 l/min, nebulizer pressure 50 psi, sheath gas temperature 350°C, sheath gas flow 12 l/min, capillary voltage 3000 V. The MS was operated in the positive ion mode using Agilent MassHunter software in the dynamic MRM (multiple reaction monitoring) mode. For quantification, a combination of external and internal calibration was applied as described previously (20).

### FICC-Seq

Full FICC-Seq methodology is available in the Supplementary Methods. The experiments were performed by immuoprecipitating endogenous TRMT2A. Briefly, HEK293 or HAP1 cells were treated with 100 μM 5-Fluorouracil (Sigma) for 24 h and then harvested for the TRMT2A-FICC-Seq experiments. The cell pellets were disrupted in lysis buffer consisting of 50  mM Tris–HCL pH 7.4, 100 mM NaCl, 1% NP-40, 0.1% sodium dodecyl sulphate, 0.5% sodium deoxycholate and treated with Turbo DNase (AM2239). Lysates were also treated with a low concentration of RNaseI (Thermofisher AM2295 at 1:200 dilution) in order to partially fragment RNAs. Lysates were cleared by centrifugation at 13 000  r.p.m. for 15 min at 4°C and then incubated with Protein G Dynabeads (Life Technologies) in the presence of mouse monoclonal anti-TRMT2A antibody (Origene, clone OTI1C3, catalog# TA505555). Following stringent washing, 3′-end dephosphorylation was performed with T4 PNK (New England Biolabs) before addition of a preadenylated linker using RNA ligase (New England Biolabs). 5′-end labelling was then performed using T4 PNK and ^32^P-ATP before protein–RNA complexes were eluted and run on Bis-Tris polyacrylamide gels. Next, nitrocellulose transfer was performed and the radioactive signal was used to dissect nitrocellulose pieces that contained TRMT2A–RNA complexes. RNA was recovered by incubating the nitrocellulose pieces in a buffer containing Proteinase K and 3.5 M urea. Reverse transcription was performed using oligonucleotides containing randomized barcodes (UMIs) and two inversely oriented adaptor regions separated by a BamHI restriction site. cDNAs were size-purified on TBE-Urea gels before being circularized by CircLigase II (Epicentre). Circularized cDNAs were then annealed to an oligonucleotide complementary to the BamHI site and then BamHI digested. Linearized, adapted cDNAs were then polymerase chain reaction (PCR)-amplified using Solexa primers with 25 cycles of PCR. Libraries containing the in-line barcodes were then sequenced on the Illumina HiSeq4000 platform.

### iCLIP

iCLIP was performed as previously described using a UV-crosslinking based method (19). The experiments were performed by immuoprecipitating endogenous TRMT2A, and the anti-TRMT2A antibody (Origene, clone OTI1C3, catalog# TA505555) used was the same as that used for TRMT2A FICC-Seq experiments. Libraries containing in-line barcodes were sequenced on the Illumina HiSeq4000 platform.

### methylation-iCLIP

For methylation-iCLIP experiments, a TRMT2A construct harbouring the CBM point mutation (Supplementary Figure S1) and an in-frame FLAG-epitope tag was used. Exponentially growing cells were transfected with TRMT2A-CBM using Lipofectamine2000, and cells were harvested 24 h later. Methylation-iCLIP was subsequently performed as described previously (6), and using an anti-FLAG antibody to immunoprecipitate ectopically expressed TRMT2A. Prepared libraries containing in-line barcodes were sequenced on the Illumina HiSeq4000 platform.

### Computational analysis

Although the automated computational pipeline iCount was originally designed for the analysis and annotation of UV-induced peptide-RNA crosslinks, we have previously shown that it is just as informative for characterizing catalytically induced peptide-RNA crosslinks (6,18). All sequencing data was processed using the iCount web server (http://icount.biolab.si) based on the iCount python package (https://github.com/tomazc/iCount). Briefly, random unique molecular identifiers were used to distinguish and discard PCR duplicate reads, and adaptor/barcode sequences were then removed (19). Trimmed reads were mapped to hg19/GRCh37 (ENSEMB v.59 GRCh37) with bowtie (v.0.12.7) using parameters ‘-v 2 -m 2 -a –best -strata’ (21), and only uniquely mapping reads were used for further analysis. iCount generated a list of significant crosslinks based on false discovery rate (FDR) <0.05 by assessing the enrichment of crosslinked nucleotides at specific sites compared to 100 random permutations (22). A peak calling algorithm was next employed to refine clusters to their most over-represented crosslink positions (local maxima). For each cluster the maximal crosslink sites were selected using a custom python script employing the peakutils package (https://pythonhosted.org/PeakUtils/index.html) with default parameters. The maximal cluster positions or ‘peaks’ were then merged across replicates to generate a final cluster list for each experiment. Counts were calculated based on all overlapping reads for each cluster per replicate using Bedtools (v2.22). These counts were normalized and processed using the DESeq2 package which included pre-filtering of low count clusters, estimation of size factors, and dispersion for library normalization. Size factors are obtained based on the ratios of total reads per sample whilst dispersions are estimated using expected mean values from the maximum likelihood estimate of log2 fold changes. Finally a generalized linear model is applied using a negative binomial distribution to identify significant changes in cluster counts between conditions. For the detection of enriched motifs associated with target bases, sequences flanking the crosslink sites (50 bp upstream and downstream) were extracted using BEDtools v.2.27.0 and run in MEME-ChIP for motif discovery and search. MEME-ChIP was run with reverse complement scans disabled and centrimo in local mode. Sequences matching the top discovered motif generated (*E*-value < 0.05) were further filtered to retain the matches with the most common position (±1) containing the crosslink site. Final cropped alignments were converted to motifs using the R packages ggplot and qqseqplot. Peak position count correlations (Pearson) within and between experiments were obtained using R and plotted with PerformanceAnalytics (https://github.com/braverock/PerformanceAnalytics) or the R package corrplot. Comparisons of peak position distribution across experiments were conducted and plotted using InteractiVenn and the R package eulerr. The tRNA structural consensus was built from the tRNAscan-SE v.2.0 results available at GtRNAdb, with nucleotide counts binned relative to the structural region they belonged to (23). Introns were removed and defining regions (loops and stems) identified using custom scripts. The final consensus region lengths and sequence were determined by average and max frequency.

## RESULTS

### TRMT2A is the major human m5U RNA methyltransferase, and is amenable to catalytic crosslinking-type experiments

Mammalian TRMT2A displays only modest sequence homology to the established yeast m5U methyltransferase Trm2. In order to determine whether human TRMT2A does indeed truly represent a cellular m5U methylase, we utilized two independent CRISPR-Cas9 mediated TRMT2A knockout cell lines (Figure 1B) for relevant mass spectrometry analysis. Strikingly, in both the HAP1 and HEK293 TRMT2A knockout cell lines, the majority of m5U signal present in total RNA from control cell lines was absent (Figure 1C). The observations suggest that TRMT2A is the major human m5U methyltransferase.

We next determined the feasibility of 5FU-mediated crosslinking to identify enzymatic RNA targets of TRMT2A. We began by performing cellular viability assays, which showed that a 24 h treatment of a 100 μM 5FU concentration inhibited cell proliferation only to a very limited extent (Supplementary Figure S1), and these conditions were used for all subsequent experiments. The 5FU treatment readily induced crosslinking between endogenous TRMT2A and RNA (Figure 1D). The yeast Trm2 m5U methyltransferase contains a key cysteine residue within its catalytic domain known to be directly critical for the methyl transfer process (13). In order to confirm that 5FU-mediated TRMT2A-RNA crosslinking was dependent on the catalytic activity of TRMT2A, we used an ectopically expressed TRMT2A construct carrying a Cys-to-Ala mutation of the corresponding conserved key catalytic nucleophile residue (catalytic nucleophile mutant, CNM) (see Supplementary Figure S2); this indeed resulted in loss of the observed TRMT2A–RNA complex formation following 5FU exposure (Figure 1E). In parallel to FICC-Seq, we also performed iCLIP and methylation-iCLIP experiments; both of these methods were anticipated to be informative in assessing the robustness of FICC-Seq to determine enzymatic target sites, as iCLIP detects only nucleotide-resolution binding sites of a protein whereas miCLIP further informs on actual methylation target sites. For iCLIP, UV-exposure was employed to induce crosslinking between endogenous TRMT2A protein and RNA (Supplementary Figure S3). For miCLIP experiments we referred to alignment of bacterial, yeast and human m5U methylases (Supplementary Figure S2). The relevant glutamate residue shown to be required for enzymatic proton abstraction from the target nucleotide (15) and thus subsequent resolution of the m5U methylase-RNA substrate covalent catalytic intermediate (24), was mutated to alanine in TRMT2A (catalytic base mutant, CBM), and this point mutant construct was ectopically expressed. This indeed promoted stabilization of TRMT2A-RNA crosslinked intermediate complexes (Supplementary Figure S3), thus indicating TRMT2A miCLIP feasibility.

### FICC-Seq demonstrates cytosolic tRNAs to be the major enzymatic targets of TRMT2A

**Figure 2.**
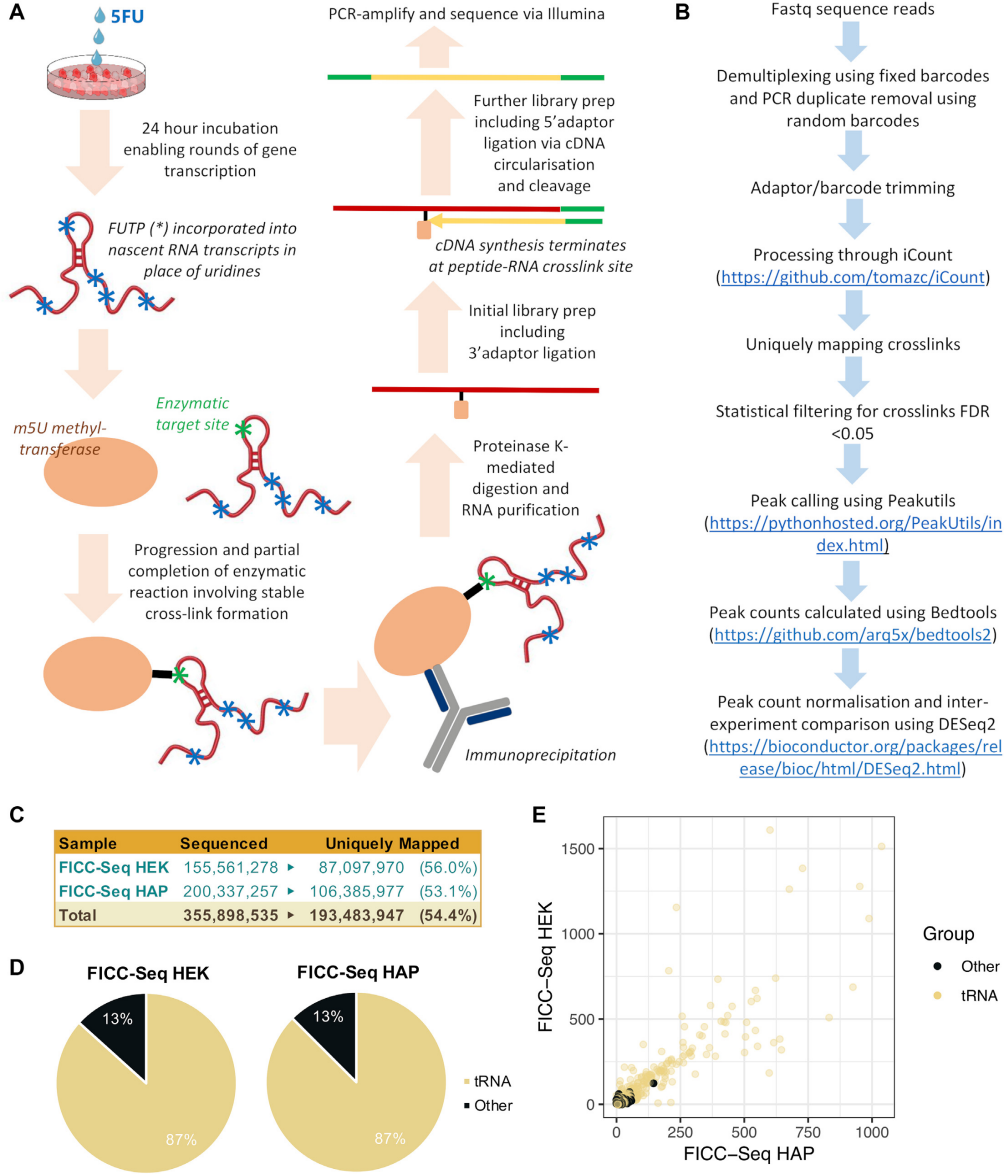
The FICC-Seq methodology identifies cytosolic tRNAs as the major enzymatic target of TRMT2A. (**A**) Summary of FICC-Seq wet-laboratory methodology. (**B**) Summary of computational analysis pipeline for sequence reads. (**C**) Numbers of total Illumina reads obtained from FICC-Seq experiments, along with the numbers of uniquely mapping reads used for downstream analyses. (**D**) Pie charts showing proportion of FICC-Seq reads mapping to tRNA (these consisted exclusively of cytosolic tRNAs, Supplementary Table S1) and non-tRNAs (Other). (**E**) Scatterplot of crosslink peak counts detected from FICC-Seq-HEK293 versus FICC-Seq-HAP1 experiments.

TRMT2A FICC-Seq experiments were performed from both HEK293 and HAP1 cells, and a summary of the overall methodology is illustrated in Figure 2A and B. Only around half of the obtained sequence reads mapped uniquely to the genome (Figure 2C), which was probably largely due to the presence of a significant proportion of reads likely mapping to multiple tRNA genes. The majority of uniquely mapping crosslink peaks indeed occurred on cytosolic tRNA genes (Figure 2D), and this observation was recapitulated by iCLIP (HEK293 and HAP1) and miCLIP (HEK293) experiments (Supplementary Table S1). The small number of crosslink peaks occurring on non-tRNA genes were further characterized by comparatively low counts (Figure 2E and Supplementary Table S1); such minority sites require validation via orthogonal biochemical methods.

**Figure 3.**
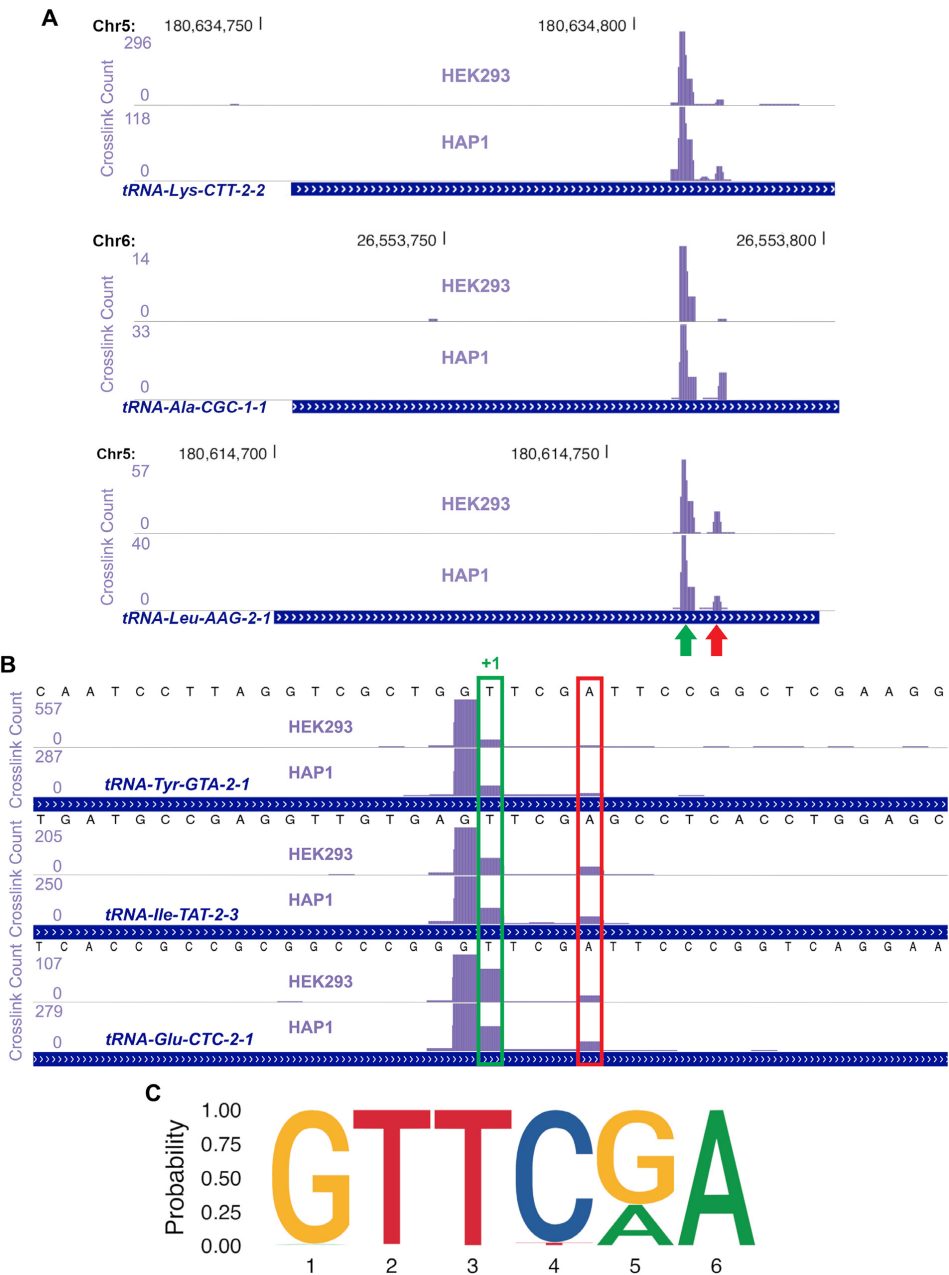
FICC-Seq reveals that TRMT2A enzymatically targets U54 within the T-loop of cytosolic tRNAs. (**A**) FICC-Seq data were viewed as custom tracks in the UCSC browser with crosslink peaks passing a FDR < 0.05 threshold annotated via our automated UV-iCLIP processing and analysis pipeline, iCount, and a few typical examples of tRNA hits are shown here. In these low-zoom views, the full tRNA genes are included. The purple bars represent the relative crosslink site density for the target region included in the view. Along the lengths of tRNAs, just a single major peak was consistently observed (green arrow), although a common sub-peak was also present (red arrow). (**B**) Higher-zoom views of a few typical tRNA hits from the TRMT2A FICC-Seq experiments are shown. As described previously (18,19), the nucleotide immediately following the final nucleotide of UV-iCLIP cDNA reads usually represent the crosslink site in RNA molecules (due to reverse transcription stalling immediately before the crosslink). In contrast, for catalytic crosslinking experiments, the crosslinked nucleotide is more often successfully reverse transcribed before resulting in truncation, and in such cases the nucleotide immediately following the iCount-annotated major crosslink site (i.e. the +1 position) is predicted to be the actual methylated base (6,18), and these are highlighted in green here. The identified target site in each of these tRNAs corresponds to position U54 in the mature tRNA molecule. The smaller sub peak was consistently observed to occur 4 nucleotides downstream from the major crosslink site, and this position corresponds to the universally conserved A58 site in tRNAs. (**C**) In order to determine whether enrichment of sequences in the immediate vicinity of TRMT2A FICC-Seq crosslink peaks on tRNA genes were present, a motif search was performed using the motif discovery tool MEME-ChIP. A clear motif consisting of the universal tRNA T-loop motif GTTCG/AA was detected.

In order to investigate the characteristics of the tRNA crosslink peaks detected, we initially viewed hits from FICC-Seq experiments on the UCSC genome browser, and a few typical examples are shown in Figure 3A (with all replicates for these available in Supplementary Figure S4). Along the length of full tRNA genes, we consistently observed a single major peak whilst a smaller associated sub-peak was also sometimes present (Figure 3A and Supplementary Figure S4). Higher-zoom views of tRNA crosslink peaks revealed that the major peak predicted precise targeting of U54 of tRNAs, whereas the sub-peak occurred on position A58 (Figure 3B and Supplementary Figure S5) which is known to undergo universal modification to methyl-1-adenosine (m1A). As previously demonstrated, during cDNA synthesis of tRNAs, reverse transcription stalling occurs frequently at the nucleotide immediately preceding tRNA-m1A58 sites (25), which is in accordance with our observed iCount-annotated peaks occurring precisely at position A58. However, the major crosslink peaks that were observed indicate that TRMT2A consistently targets U54 of cytosolic tRNAs for modification (Figure 3B and Supplementary Figure S5). Furthermore, a striking enrichment of the universally conserved T-loop motif sequence of tRNAs was present in the FICC-Seq dataset (Figure 3C), suggesting that enzymatic targeting of tRNA-U54 by TRMT2A was likely common.

### FICC-Seq is a particularly robust method for nucleotide-resolution detection of TRMT2A enzymatic target sites

**Figure 4.**
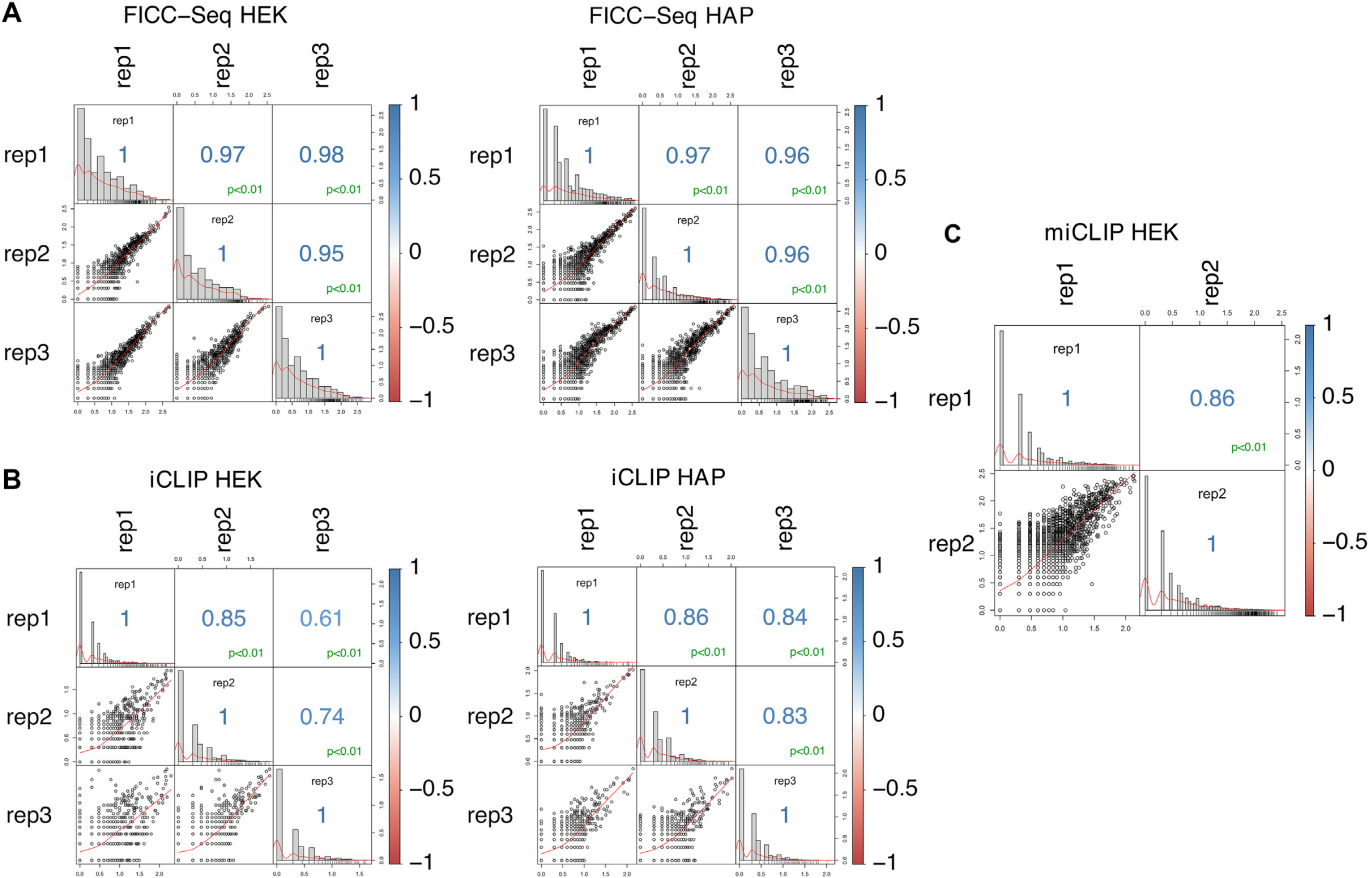
FICC-Seq presents the most robust crosslink occurrence reproducibility. Intra-experimental comparison of the number of crosslinks counts detected per peak (FDR < 0.05). Each matrix reports Pearson's correlation coefficients and corresponding log transformed scatterplots between biological replicates.

**Figure 5.**
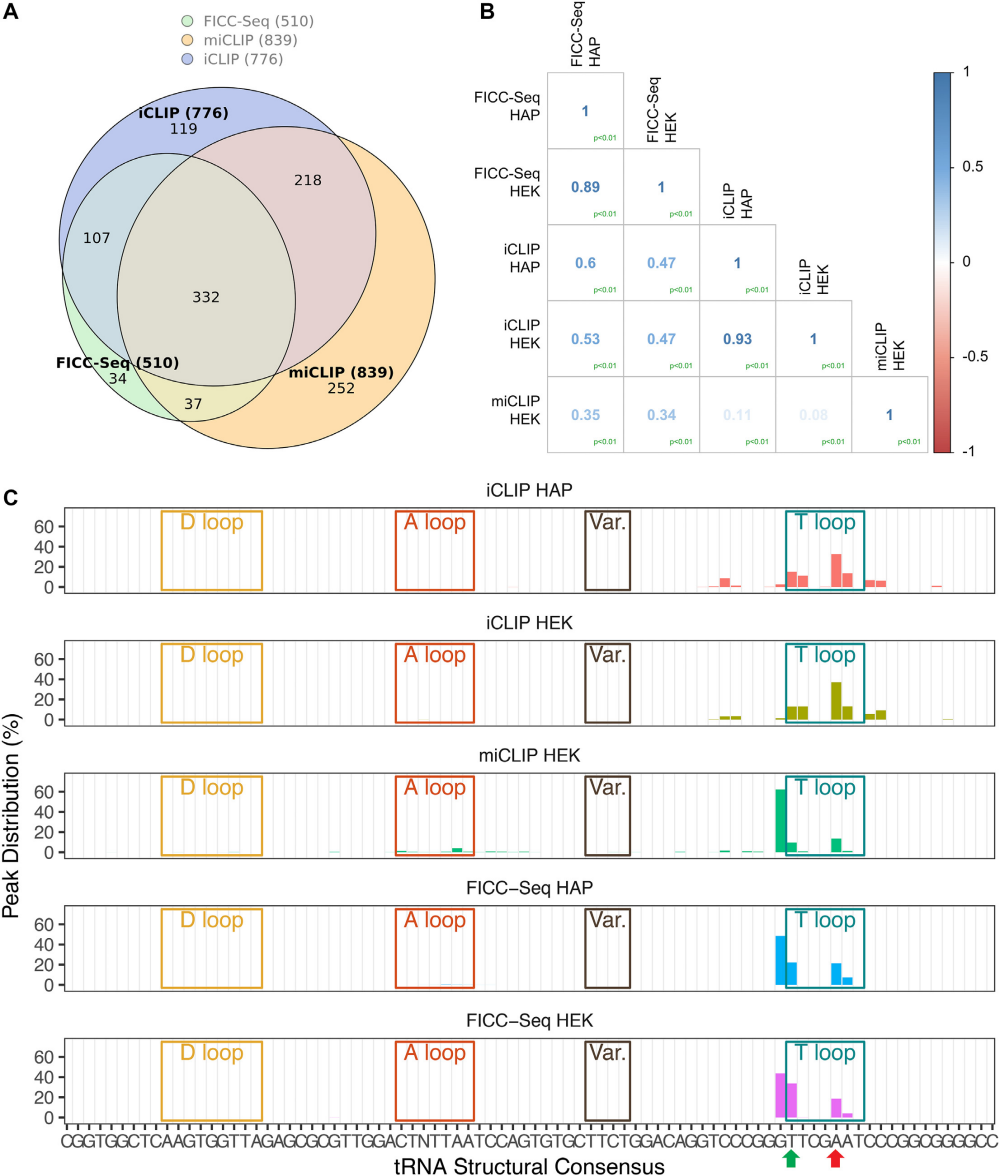
FICC-Seq displays the highest accuracy for TRMT2A enzymatic target-site detection. (**A**) Venn diagram depicting overlap of genomic crosslink peak positions (FDR < 0.05) detected by FICC-Seq, miCLIP and iCLIP experiments. Only peaks with a minimum of five crosslink counts are included. (**B**) Matrices showing inter-experimental comparison of crosslink peak position counts (FDR < 0.05) via Pearson's correlation coefficients. Counts for each experiment were grouped from replicates and normalized as described in the ‘Materials and Methods’ section. (**C**) For each experiment type, all detected crosslinks peaks (FDR < 0.05) mapping to tRNAs were compiled in order to gain an overview of targeted positions along mature tRNA molecules. Defining secondary structure regions were identified from tRNAscan-SE results (see ‘Materials and Methods’ section) to form the tRNA structural consensus. tRNA crosslink site positions were converted relative to the relevant tRNA region they resided in and subsequently plotted on the consensus. For UV-iCLIP experiments (top two panels), multiple crosslink peaks flanking the U54 position were present, suggesting multiple TRMT2A-tRNA contact points during the methylation process. For the miCLIP (middle panel) and FICC-Seq (bottom two panels) catalytic crosslinking experiments, a predominant crosslink peak is present which predicts methylation precisely at position U54 of the tRNA consensus (green arrow). Whilst the expected sub-peak at position A58 corresponding to the m1A modification site (red arrow) can also be observed in FICC-Seq experiments, there is absence of the off-target minor peaks that are otherwise observed in miCLIP experiments.

To determine the robustness of the FICC-Seq method, we initially investigated the correlation of genomic crosslink peak position counts obtained between intra-experimental biological replicates; better correlations for the FICC-Seq experiments in comparison to iCLIP and miCLIP experiments were consistently observed (Figure 4). Indeed, although there was good overlap of crosslink peak positions detected between the different types of experiment (Figure 5A and Supplementary Figure S6), inter-experimental correlation of the crosslink peak position counts was sometimes poor (Figure 5B and Supplementary Figure S7). Nonetheless, the fact that 93% of the TRMT2A target sites detected by FICC-Seq were still cross-validated by either miCLIP and/or iCLIP experiments (Figure 5A) indicates a low false detection rate of the method.

To investigate the seeming disparity of crosslink peak positions detected by the different methods further, we compiled all of the tRNA crosslink peaks present in each dataset and plotted them along the length of the full consensus tRNA sequence (Figure 5C). iCLIP tRNA hits displayed crosslink peaks in and around the T-loop suggesting that there may be multiple contact points between TRMT2A and tRNAs during the methylation process (Figure 5C, top two panels). Although the likely m1A peak at position 58 was present, methylation-iCLIP demonstrated enzymatic targeting of position U54 by TRMT2A (Figure 5C, middle panel); however, minor crosslink peaks across some of the length of the tRNA, including in the anticodon loop (A loop), were also observed (Figure 5C, middle panel). As m5U has never been observed at these additional tRNA positions in previous studies, these were most likely a result of off-target detections caused by ectopic expression of TRMT2A in this particular experiment. In support of the view that the minor crosslink peaks detected by miCLIP did not represent true methylation targets of TRMT2A, we failed to observe these in the FICC-Seq experiments. FICC-Seq indeed instead displayed a clean detection of targeting occurring commonly and exclusively at position U54 of tRNAs (Figure 5C, bottom two panels).

**Figure 6.**
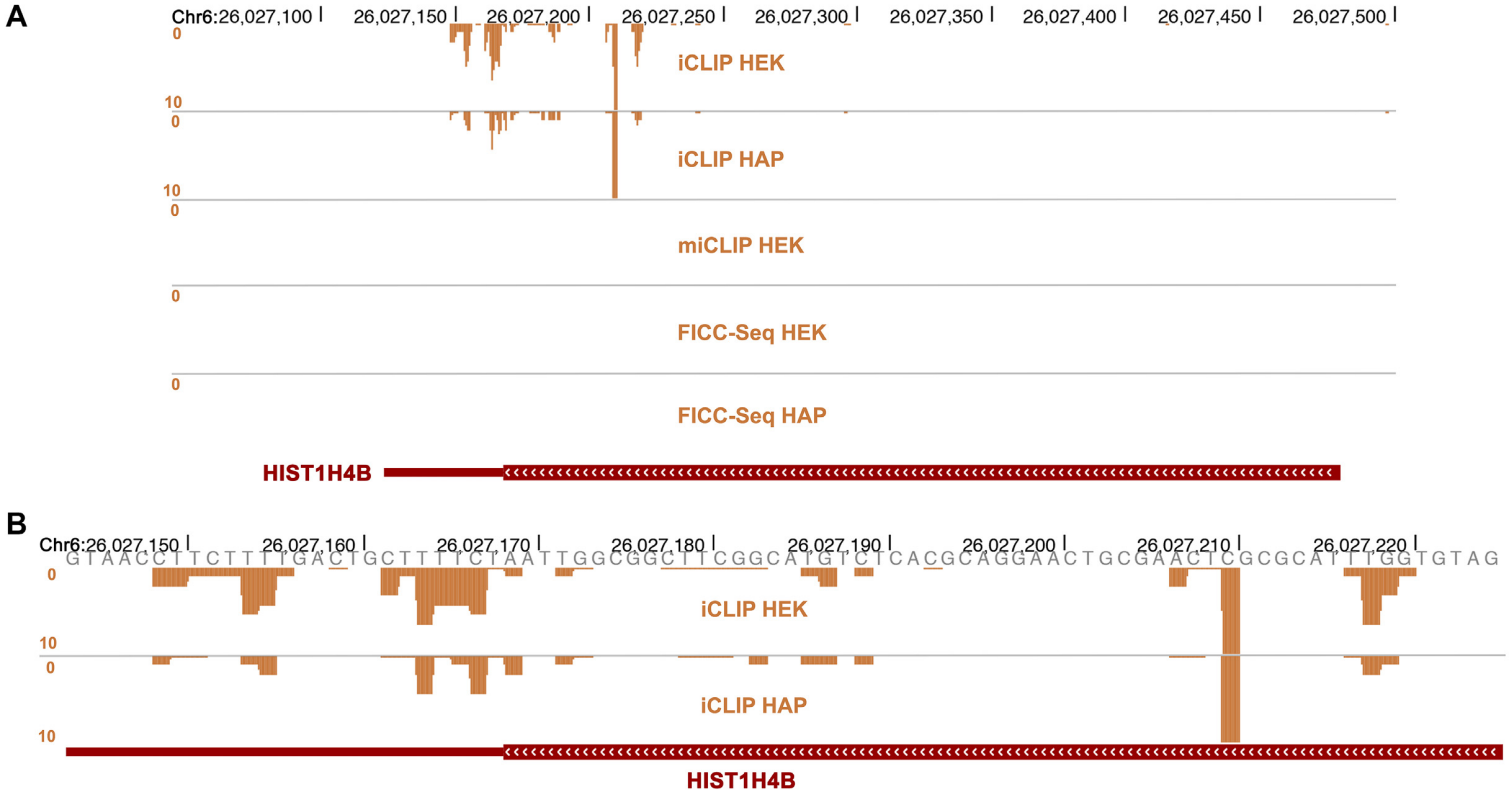
TRMT2A binds to multiple sites in the HIST1H4B messenger RNA but does not target them for enzymatic modification. (**A**) Crosslinks (FDR < 0.5) occurring on the *HIST1H4B* gene from each of the five experiments performed in this study are shown. The *HIST1H4B* gene occurs on the negative DNA strand (hence the negative-oriented peaks), is ∼300 nt in length and contains no introns; the full gene is included in the view. The iCLIP experiments show that TRMT2A binds to sites in the 3′coding sequence as well as in the 3′UTR. No crosslinks were detected in the miCLIP or FICC-Seq experiments for this gene. (**B**) High-zoom view of (A) encompassing only the crosslink sites from the iCLIP experiments.

A small number of TRMT2A crosslinks mapping to non-tRNA genes were detected in our experiments, although some of these were only detected by iCLIP; a prominent example is shown in Figure 6. Multiple crosslinks in the 3′ portion of the HIST1H4B transcript were observed from iCLIP-HEK and iCLIP-HAP experiments. However, these crosslinks were absent in FICC-Seq and miCLIP experiments (Figure 6), suggesting that TRMT2A stably binds the HIST1H4B messenger RNA but does not methylate it. TRMT2A has been demonstrated to be a cell cycle-regulated protein with peak expression occurring during S-phase (26–28). Similar to another cell cycle-regulated human RNA methyltransferase, NSUN2, which also has enzymatic activity-independent functional roles (29), it is quite likely that TRMT2A has biological roles that are independent of it methyltransferase activity; interaction with transcripts such as HIST1H4B messenger RNA might be related to such additional functional tasks. The potential for FICC-Seq to distinguish between RNA sites that are enzymatically targeted by a modification writer, as opposed to those that are only bound by it, further underscores the robustness of the method.

## DISCUSSION

In this study we have developed and used FICC-Seq to characterize the genome-wide targets of the TRMT2A methyl-5-uridine writer in human cellular RNA. Our findings reveal that TRMT2A is the major human m5U methyltransferase and that it commonly targets U54 of cytosolic tRNAs. Although the primary functions of m5U54 in cytosolic tRNA remain to be determined in detail, it has recently been shown to be involved in the Toll-like receptor 7-dependent immune response (30). By establishing TRMT2A as the human genetic factor likely solely responsible for cytosolic m5U54 formation, our study should aid further investigations into the regulation of the modification and its biological functions.

Although our mass spectrometry experiments demonstrate that TRMT2A is responsible for the majority of m5U present in cellular RNA, given the high similarity of the predicted catalytic domain of TRMT2B with that of its paralogue, it is probable that it too represents an m5U methyltransferase. A previous study has demonstrated TRMT2B as a mitochondrial protein (31), and it has been proposed that it may represent a mitochondrial tRNA m5U54 methyltransferase (32). Mitochondrial tRNAs are non-canonical particularly in terms of their structure, often for example lacking a T-loop (33), and it has accordingly been shown that the corresponding cytosolic m5U54 is in fact sparse in mammalian mitochondrial tRNAs (34). Indeed, tRNA m5U54 has thus far only been shown to be present in two human mitochondrial tRNAs, Leu-UUR (35) and Ser-UCN (36). It is thus possible that whilst TRMT2A commonly targets cytosolic tRNA-U54, TRMT2B might instead represent a mitochondrial tRNA m5U54 methyltransferase with a limited number of actual targets; appropriate TRMT2B FICC-Seq experiments potentially should be capable of characterizing this further. It is likely that the FICC-Seq method could also be applied to profile targets of specific pseudouridine synthases based on catalytic crosslinking principles (8), given that the catalytic mechanism of some of these also proceeds via covalent intermediate formation. For example, similar to m5U methyltransferases, some bacterial pseudouridine synthases, such as TruA and RluA, have also been shown to form stable covalent complexes during the catalytic process when incubated with tRNA substrates containing 5FU in place of target uridines (37–39). As the catalytic mechanisms of pseudouridine synthases is thought to be evolutionarily conserved, at the least within phylogenetic families, TruA- and RluA-mammalian homologs may also be amenable to enzyme-specified profiling of the pseudouridine modification via the method.

We have observed significant *in vivo* TRMT2A-RNA crosslinking following only reasonable doses of cellular 5FU exposure in our study, which potentially has important biological implications. For example, it has been shown that in addition to inhibition of thymidylate synthase, uncharacterized RNA-mediated pathways very likely also play significant roles in the cytotoxic mechanism of 5FU (40,41). It is therefore of interest that whilst disruption of pseudouridine synthase or dihydrouridine synthase, as well as pseudouridine metabolizing enzymes in yeast leads to hypersensitivity to 5FU, disruption specifically of the TRMT2A homologue instead leads to some resistance to the compound (42). It is possible that TRMT2A-RNA crosslinking upon 5FU exposure represents a tangible relevant pathway contributing to the cytotoxic effect of the drug. A more complete understanding of such additional cytotoxicity mechanisms of 5FU, an important chemotherapeutic agent, could conceivably aid in modulating its efficient use.

## DATA AVAILABILITY

All source sequencing data is available from the NCBI repository under GEO accession: GSE109183.

## Supplementary Material

gkz658_Supplemental_Files
