# Supplementary material for: FICC-Seq: a method for enzyme-specified profiling of methyl-5-uridine in cellular RNA
Source: Nucleic Acids Res. 2019 Jul 30;47(19):e113. doi: 10.1093/nar/gkz658 (PMC6821191; doi:10.1093/nar/gkz658)
Supplement: gkz658_Supplemental_Files [file gkz658_supplemental_files.zip › Supplementary Methods.docx]

**Supplementary Methods**

*Fluorouracil Induced Catalytic Crosslinking-Sequencing (FICC-Seq)*

Exponentially growing HEK293 or HAP1 cells were treated with 100µM 5-Fluorouracil (Sigma) for 24 hours and harvested. Cell pellets were then disrupted by intermittent pipetting in 0.8ml lysis buffer (50 mM Tris-HCL pH 7.4, 100 mM NaCl, 1% NP-40, 0.1% SDS, 0.5% sodium deoxycholate) and incubated at 4°C for 30 minutes. Lysates were next treated with 2µl Turbo DNase (AM2239) and a low concentration of RNaseI (2 µl of Ambion AM2295 diluted 1:200) at 37°C for 3 minutes in order to degrade DNA and only partially fragment RNAs, and then transferred immediately to wet ice for 5 minutes. Lysates were then cleared by centrifugation at 13,000 r.p.m. for 15 minutes at 4°C and then stored on wet ice until needed. Meanwhile, 100 µl of Protein G Dynabeads (Life Technologies) was washed 3 times in 1ml lysis buffer and incubated with 0.5ml lysis buffer in the presence of 5µg anti-TRMT2A antibody (Origene, OTI1C3, Catalog# TA505555) for 30 minutes at 4°C. Protein G-antibody complexes were then washed 3 times in 1ml lysis buffer and resuspended in the clarified cell lysate; immunoprecipitations were carried out for 2 hours at 4°C with constant gentle rotation. The Dynabeads were then washed 5 times with 1ml lysis buffer and twice with 1ml PNK wash buffer (20 mM Tris-HCl pH 7.4, 10 mM MgCl_2_, 0.2 % Tween-20). RNA 3’end dephosphorylation was then carried out by resuspending the Dynabeads in the following mix: 4 µl 5xPNK pH 6.5 buffer (50 mM Tris–HCl, pH 6.5, 50 mM MgCl_2,_ 5 mM dithiothreitol), 0.5 µl polynucleotide kinase (PNK) (NEB), 0.5 µl RNase Inhibitor (Invitrogen), 15 µl H_2_0; the mix was incubated at 37°C for 20 minutes. Dynabeads were then washed three times with PNK wash buffer before proceeding to the RNA linker ligation, which was carried out by resuspending the Dynabeads in the following mix: 5 µl 4x ligation buffer (200 mM Tris–HCl, pH 7.8, 40 mM MgCl_2,_ 4 mM dithiothreitol), 1 µl T4 RNA ligase (NEB), 0.5 µl RNase Inhibitor, 1.5 µl 20 µM preadenylated adapter L3-App (rAppAGATCGGAAGAGCGGTTCAG/ddC/), 4 µl PEG400, and incubating overnight at 16°C in a thermomixer set at 1100 r.p.m. Dynabeads were then washed three times in PNK wash buffer before proceeding to 5’end phosphorylation/labelling of RNAs which was carried out by resuspending the Dynabeads in the following mix: 1 µl PNK (NEB), 2 µl 10x PNK buffer (NEB), 2 µl gamma-P32-ATP (Perkin Elmer), 15 µl H_2_0, and incubating at 37°C for 5 minutes. The supernatant was then removed from the beads which were then resuspended in 20 µl of 1x NuPAGE loading buffer (Invitrogen), and heated to 70°C for 5 minutes. The eluate was then loaded onto Novex NuPAGE 4-12% Bis-Tris gels (Invitrogen), and electrophoresis performed in the presence of 1× MOPS running buffer (Invitrogen). Protein-RNA complexes were next transferred to a nitrocellulose membrane (Whatman) using the Novex wet transfer apparatus (Invitrogen). The nitrocellulose membrane was then exposed to a BioMax XAR Film (Kodak) in order to locate regions containing labelled protein-RNA complexes, which were then cut from the membrane using a sharp scalpel. For retrieval of crosslinked RNAs, the nitrocellulose pieces were incubated in a 200µl mix consisting of: 200µg proteinase K, 100 mM Tris-HCl pH 7.5, 50 mM NaCl, 10 mM EDTA for 20 minutes at 37°C in a thermomixer set at 1,100 r.p.m. Next, 200µl of the following mix: 100 mM Tris-HCl pH 7.5, 50 mM NaCl, 10 mM EDTA, 7 M urea, was added, and the samples were incubated for a further 20 minutes. The 400µl solution containing digested protein and eluted RNAs was then added to a 2ml Phase Lock Gel Heavy tube (5 prime), to which 400µl of phenol/chloroform (Ambion) was also added. The tube was incubated at 30°C in a thermomixer set at 1100 r.p.m for 5 minutes, before separating the phases by centrifuging at 13,000 r.p.m for 5 minutes at room temperature. The aqueous phase was transferred to a 1.5ml Eppendorf tube and 1µl glycoblue, 40µl 3M sodium acetate pH5.5, 1ml 100% ethanol added before placing at -20°C overnight to allow precipitation. RNAs were then pelleted by centrifugation for 30 min at 15,000 r.p.m and 4°C, washed with 1ml 80 % ethanol and resuspended in 5µl H_2_O. 1µl of RT primer (5Phos/NNXXXXNNNAGATCGGAAGAGCGTCGTGGATCCTGAACCGC; N’s denote random nucleotides enabling identification of PCR duplicates, and X’s denote a fixed 4-nucleotide sequence enabling demultiplexing of pooled samples for Illumina sequencing) and 1µl dNTP mix (NEB) was then added to the RNA sample and incubated at 65°C for 5 minutes before snap-chilling on wet ice for 5 minutes. Next 7 µl H_2_O, 0.5 µl Superscript II, 0.5 µl RNase Inhibitor, 1 µl 0.1M dithiothreitol, 4 µl 5x First strand buffer was added, and reverse transcription performed with the following programme: 25 °C for 5 min, 42 °C for 20 min, 50 °C for 40 min, 80 °C for 5 min, then hold at 4 °C. Next, 380µl H_2_O, 1µl glycoblue, 40µl 3M sodium acetate pH5.5, 1ml 100% ethanol was added before placing at -20°C overnight to allow precipitation. cDNAs were then pelleted by centrifugation for 30 minutes at 15,000 r.p.m and 4°C, washed with 1ml 80 % ethanol and resuspended in 6µl H_2_O. For size separation, cDNAs were mixed with 2µl 2× TBE-urea loading buffer (Invitrogen) and incubated for 3 minutes at 70°C. Samples were run on a 6 % TBE urea gel (Invitrogen) in 1× TBE buffer for 40 minutes at 180 V. Bands were then cut from the gel corresponding to cDNA sizes of 70-150nt. Gel fragments were mixed with 400ml TE buffer, crushed with a 1 ml syringe plunger and incubated for 2 hours at 37°C and 1,100 r.p.m. A Costar SpinX column (Corning Incorporated) was prepared by addition of two 1 cm glass wool pre-filters (Whatman 1823-101). The gel supernatant containing the eluted cDNA was then transferred to the column and centrifuged for 1 min at 13,000 r.p.m. The flow-through was collected and added to a fresh 1.5ml Eppendorf tube, and 40µl 3 M sodium acetate pH 5.5 and 1µl glycogen and 1ml 100 % ethanol was added before incubating overnight at –20°C. The size-selected cDNAs were then pelleted by centrifugation for 30 minutes at 15,000 r.p.m and 4°C, then washed with 1ml 80 % ethanol and resuspended in 6.5 µl H_2_O. Next, cDNAs were circularised by adding 0.8µl 10× CircLigase buffer II (Epicentre), 0.4µl 50 mM MnCl_2_ and 0.3µl CircLigase II (Epicentre) to give total 8µl volume which was incubated for 1 hour at 60°C. For subsequent custom linearization, an annealing oligonucleotide complementary to a specifically-located BamHI restriction site in the RT primer was annealed by adding 26µl H_2_O, 3µl FastDigest buffer (Fermentas) and 1µl 10 µM BamHI oligo (GTTCAGGATCCACGACGCTCTTCAAAA) and incubation with the following program: 2 min at 95°C, 70 cycles starting for 1 min at 95°C and reducing the temperature with every cycle by 1°C. The BamHI cleavage was then carried out via addition of 2µl Fastdigest BamHI (Fermentas) and incubating for 30 minutes at 37°C. Samples were then mixed with 350µl TE buffer, 1µl glycoblue, 40µl 3M sodium acetate pH 5.5 and 1ml 100% ethanol and incubated overnight at –20°C. cDNAs were then precipitated by centrifugation for 30 minutes at 15,000 r.p.m and 4°C, and pellets washed with 1ml 80 % ethanol, before resuspending in 20µl H_2_O. Next, 10µl of the correctly adapted linearized cDNA was mixed with 1µl 10µM P5 Solexa PCR primer (AATGATACGGCGACCACCGAGATCTACACTCTTTCCCTACACGACGCTCTTCCGATCT), 1µl 10 µM P3 Solexa PCR primer (CAAGCAGAAGACGGCATACGAGATCGGTCTCGGCATTCCTGCTGAACCGCTCTTCCGATCT), 20µl Accuprime Supermix (Invitrogen), 8µl H_2_O, heated to 94 °C for 2 minutes and subjected to 25 cycles of PCR with the following programme: 94 °C for 15 s, 65 °C for 30 s, 68 °C for 30 s; complete with 68 °C for 3 min, then hold at 25 °C. The PCR product was then mixed with TBE loading buffer and electrophoresed on a 6% TBE polyacrylamide gel, which was then stained with SYBR green I. The visualised amplified library corresponding to sizes 150nt-200nt was then excised from the gel using a clean blade. The gel pieces were then mixed with 400ml TE buffer, crushed with a 1ml syringe plunger and incubated for 2 hours at 37°C and 1,100 r.p.m. A Costar SpinX column (Corning Incorporated) was prepared by addition of two 1cm glass wool pre-filters (Whatman 1823-101). The gel supernatant containing the eluted PCR product was then transferred to the column and centrifuged for 1 minute at 13,000 r.p.m. The flow-through was collected and added to a fresh 1.5ml Eppendorf tube, and 40µl 3M sodium acetate pH 5.5, 1µl glycogen and 1ml 100 % ethanol was added before incubating overnight at –20°C. The DNA library was pelleted by centrifugation for 30 minutes at 15,000 r.p.m at 4°C, washed with 1ml 80 % ethanol, and then resuspended in 20µl H_2_O. Final libraries were clustered and sequenced single end using 50 cycles of reversible dye terminator chemistry on an Illumina HiSeq4000 at the Cambridge Institute Genomics Core, UK.
