# Supplementary material for: FICC-Seq: a method for enzyme-specified profiling of methyl-5-uridine in cellular RNA
Source: Nucleic Acids Res. 2019 Jul 30;47(19):e113. doi: 10.1093/nar/gkz658 (PMC6821191; doi:10.1093/nar/gkz658)
Supplement: gkz658_Supplemental_Files [file gkz658_supplemental_files.zip › Supplementary_Figures_.pdf]

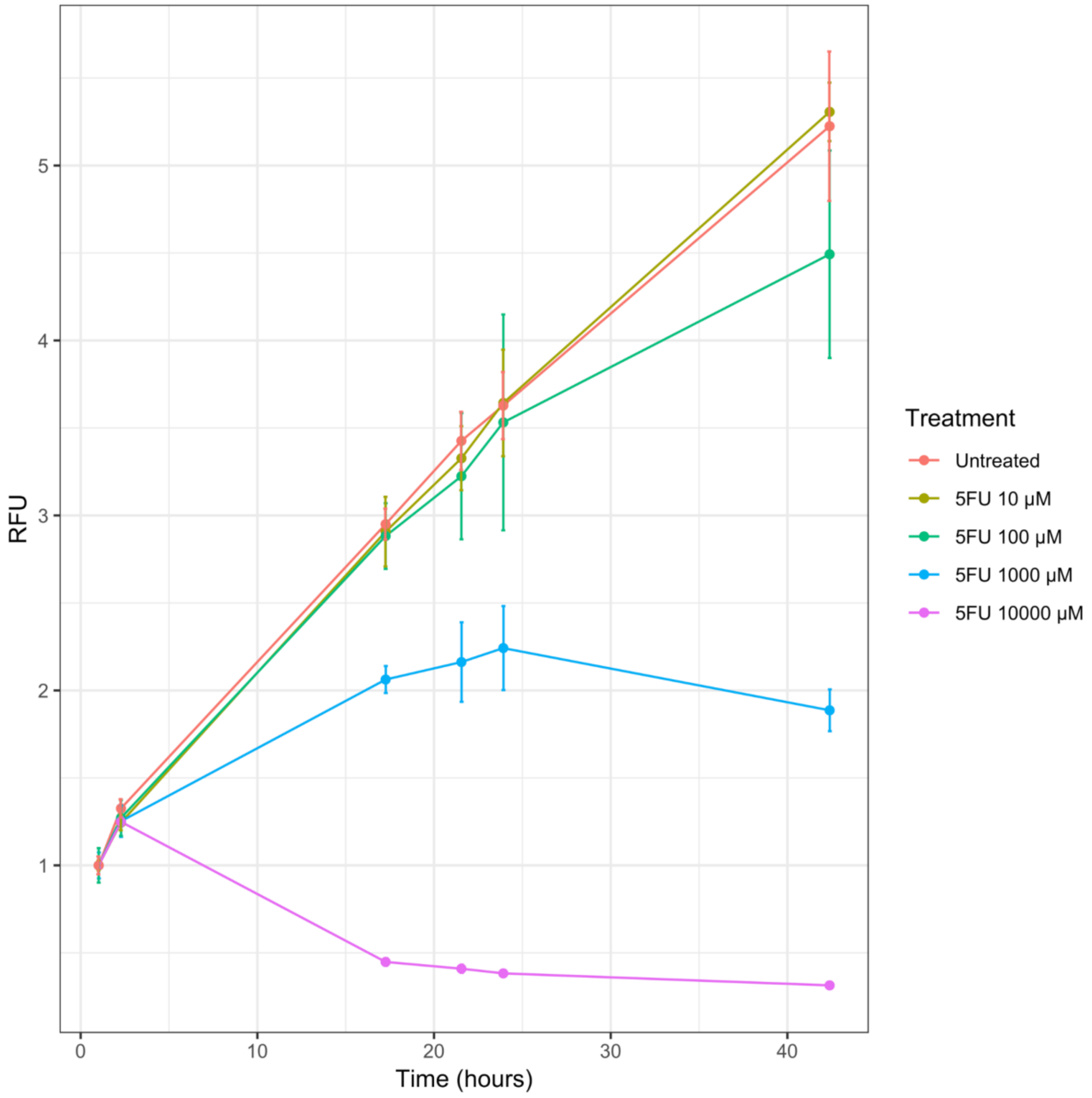

Figure S1. Cell survival following various doses of 5FU treatment of HEK293 cells. As per the RealTime-Glo MT Cell Viability Kit used (see *Materials and Methods*), RFU (Relative Fluorescence Units) is proportional to cell viability. Error bars indicate standard deviation from the average for every triplicate treatment group.

```
                                                        #
TRMT2A          ---ASQHLVAILDPPRAGLHSKVILAIRRAKNLRRLLYVSCNPRAA-MGN
TRMT2B          --EDGQSIVAVVNPARAGLHYKVIQAIRNFRAIHTLVFVSCKLHGESTRN
Trm2            ----SENTSVILDPPRKGCDELFLKQLAA-YNPAKIIYISCNVHSQ-ARD
TrmA            DLKSYQCETIFVDPPRSGLDSETEKMVQA---YPRILYISCNPETL-CKN
                     :    .::*.* * .      :        ::::**: .     :

TRMT2A          FVDLCRAP--SNRVKGIPFRPVKAVAVDLFPQTPHCEMLILFERVEHPNG
TRMT2B          VIELCCPPDPAKKLLGEPFVLQQAVPVDLFPHTPHCELVLLFT-------
Trm2            VEYFLKET--E---NGSAHQIESIRGFDFFPQTHHVESVCIMKR------
TrmA            LETLS-----------QTHKVERLALFDQFPYTHHMECGVLLTA------
                .  :             ..       .* ** * * *   ::
                                                    +
```

Figure S2. Amino acid sequence alignment of the catalytic domain of m5U methylases is shown. The four proteins included here are TrmA (*E.coli*), Trm2 (*S.cerevisiae*), and the two predicted human homologs TRMT2A and TRMT2B. The cysteine residue indicated by the hash (#) sign has been shown to be the key catalytic nucleophile, and is required for covalent bond formation with the target uridine; for generation of the TRMT2A-CNM construct, this cysteine was mutated to alanine. The glutamate residue indicated by the plus (+) sign was previously shown to likely act as the catalytic base required for proton abstraction from the modified nucleotide (14), and is thus also critical for resolving the methylase-RNA substrate covalent intermediates (23); for generation of the TRMT2A-CBM construct used for methylation-iCLIP experiments, this glutamate was mutated to alanine.

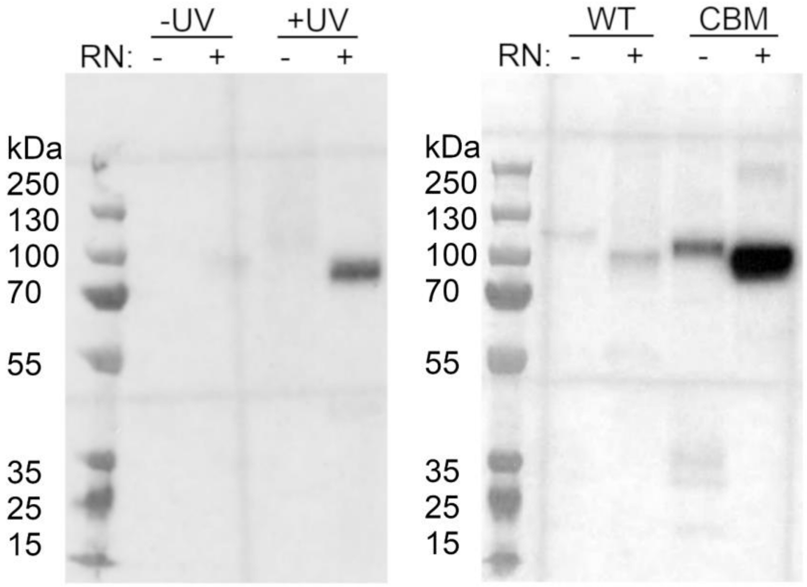

Figure S3. iCLIP feasibility experiments were performed using an anti-TRMT2A antibody for the immunoprecipitations. UV-treatment of HEK293 cells efficiently induced covalent crosslinks between endogenous TRMT2A and RNA (autoradiograph, left). Such feasibility assays were also carried out for methylation-iCLIP experiments (autoradiograph, right); TRMT2A-catalytic base mutant (CBM) (see Figure S2) was ectopically expressed, and here the appropriate control consisted of parallel ectopic expression of a TRMT2A-WT construct (WT). An anti-FLAG antibody used for the immunoprecipitations. While faint bands can be observed in the TRMT2A-WT condition, the corresponding bands observed in the TRMT2A-CBM condition were considerably stronger, thus confirming the ability of the CBM to promote efficient crosslink stabilisation.

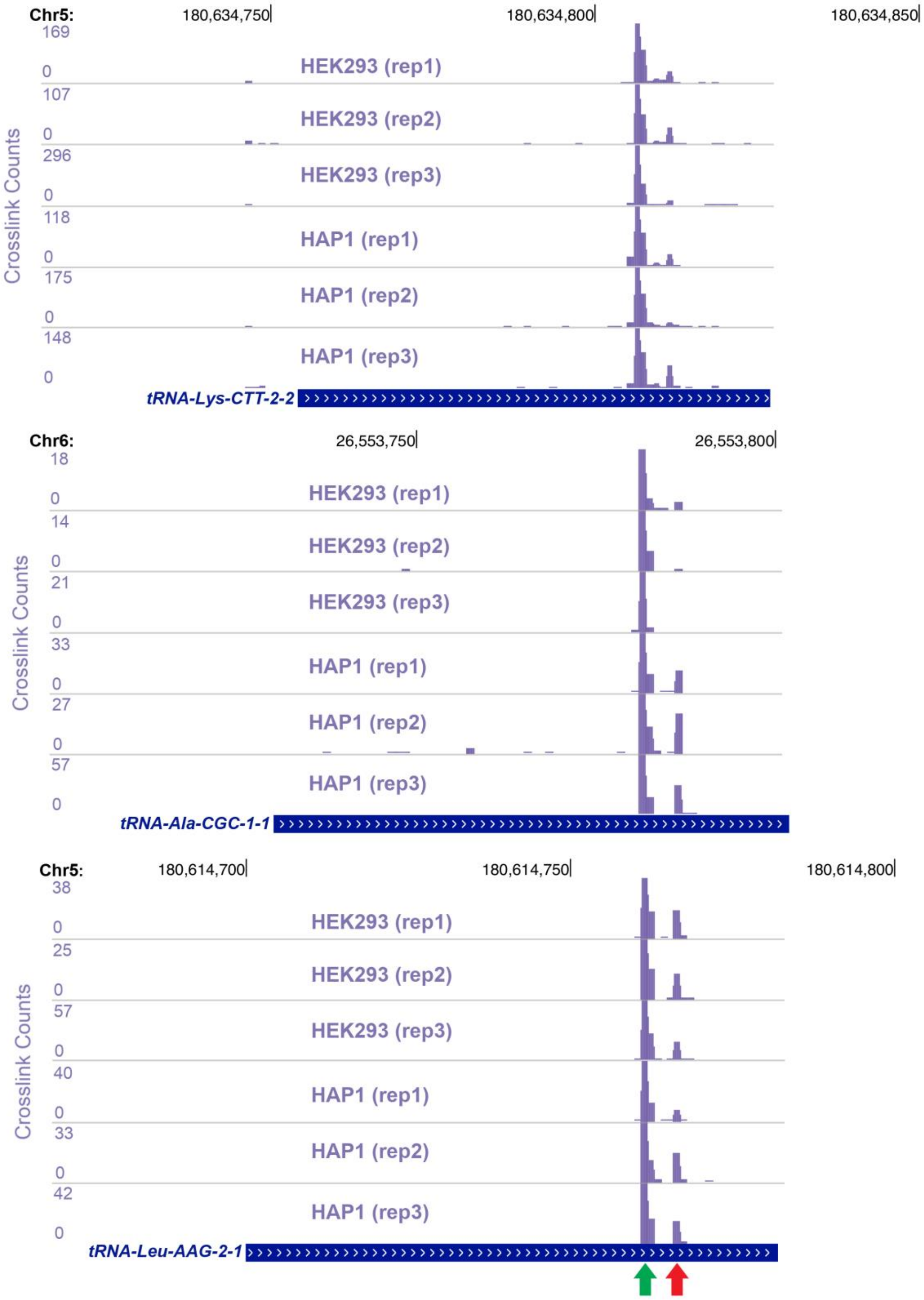

Figure S4. Browser views including all replicates of data shown in Figure 3A.

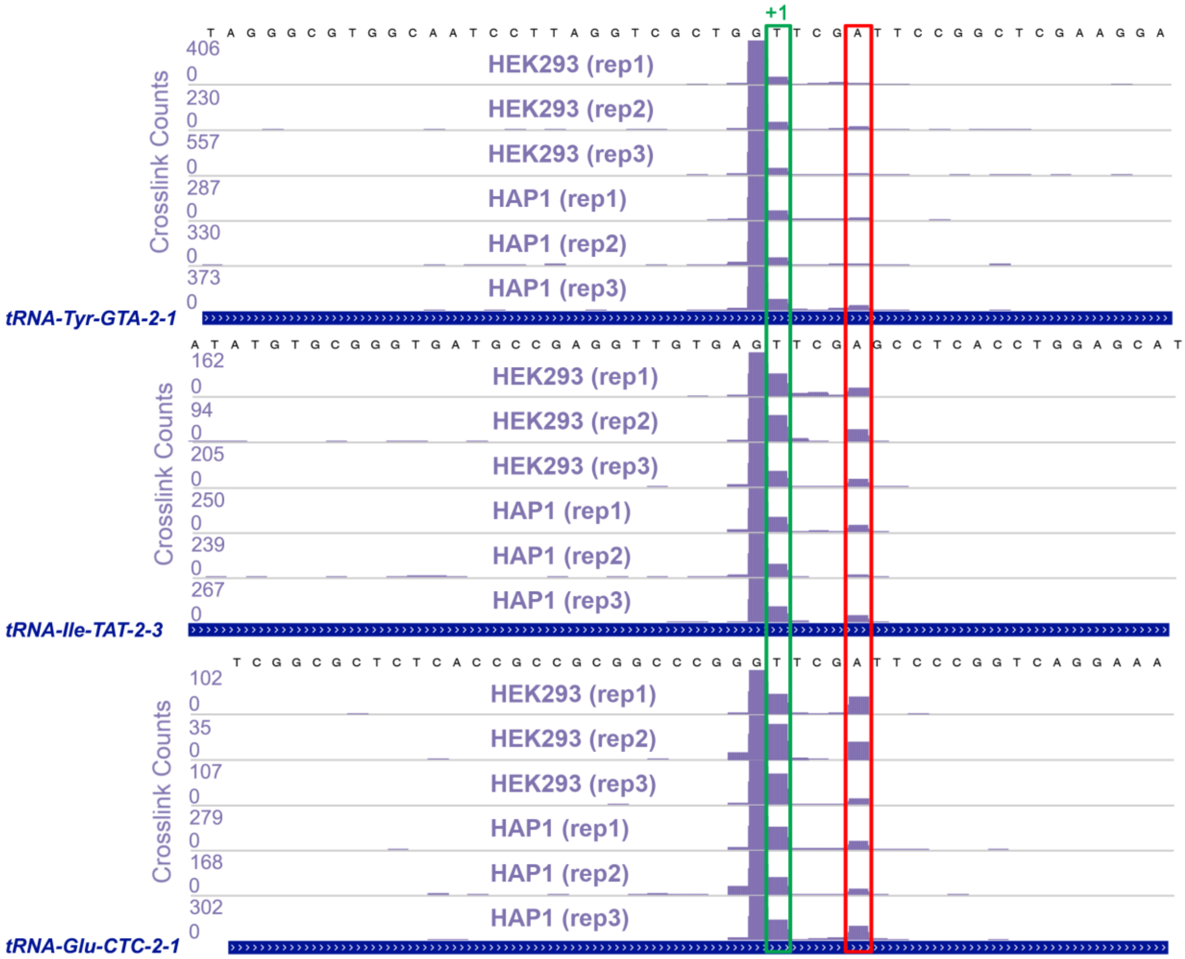

Figure S5. Browser views including all replicates of data shown in Figure 3B.

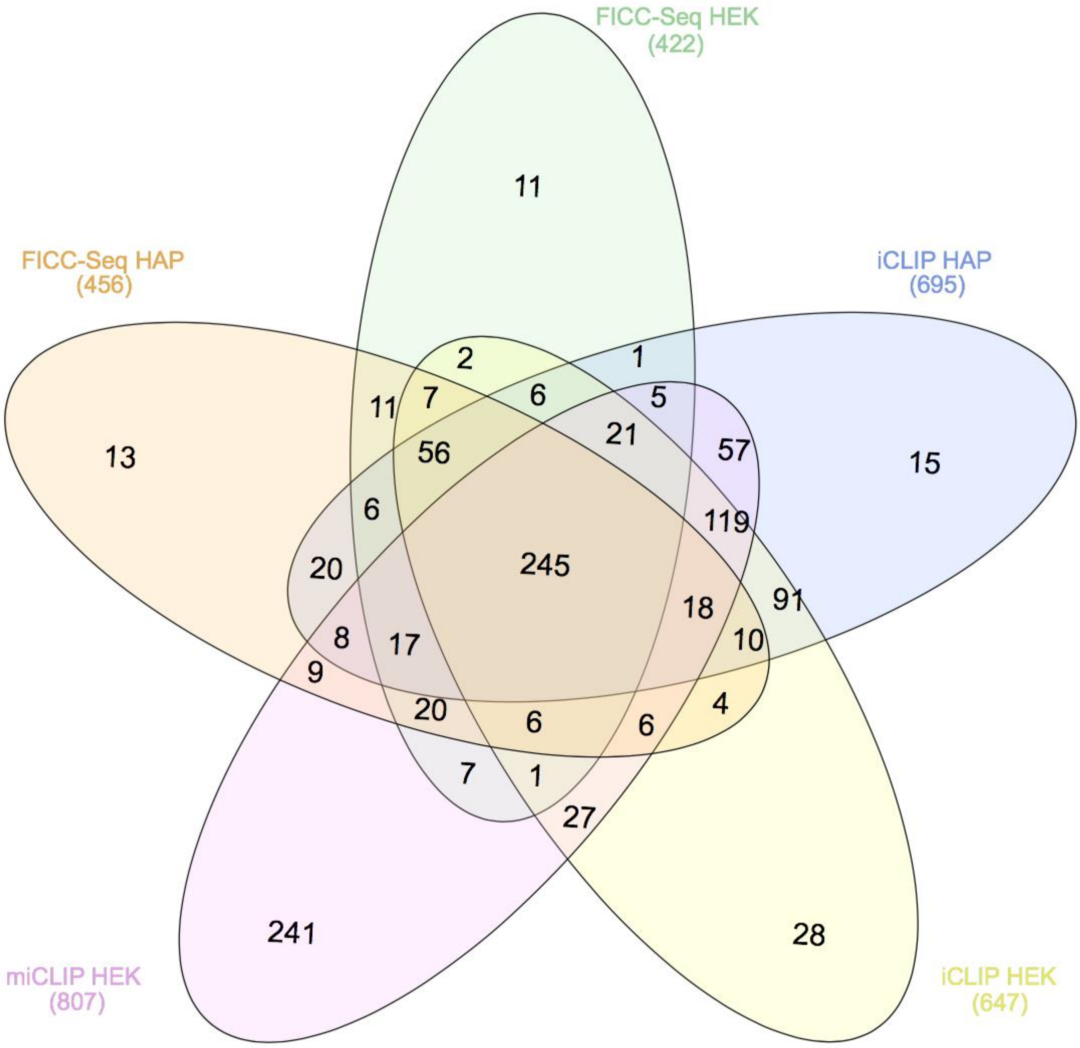

Figure S6. Venn diagram depicting overlap of genomic crosslink peak positions (FDR <0.05) detected by the different experiments. Only peaks with a minimum of 5 crosslink counts are included.

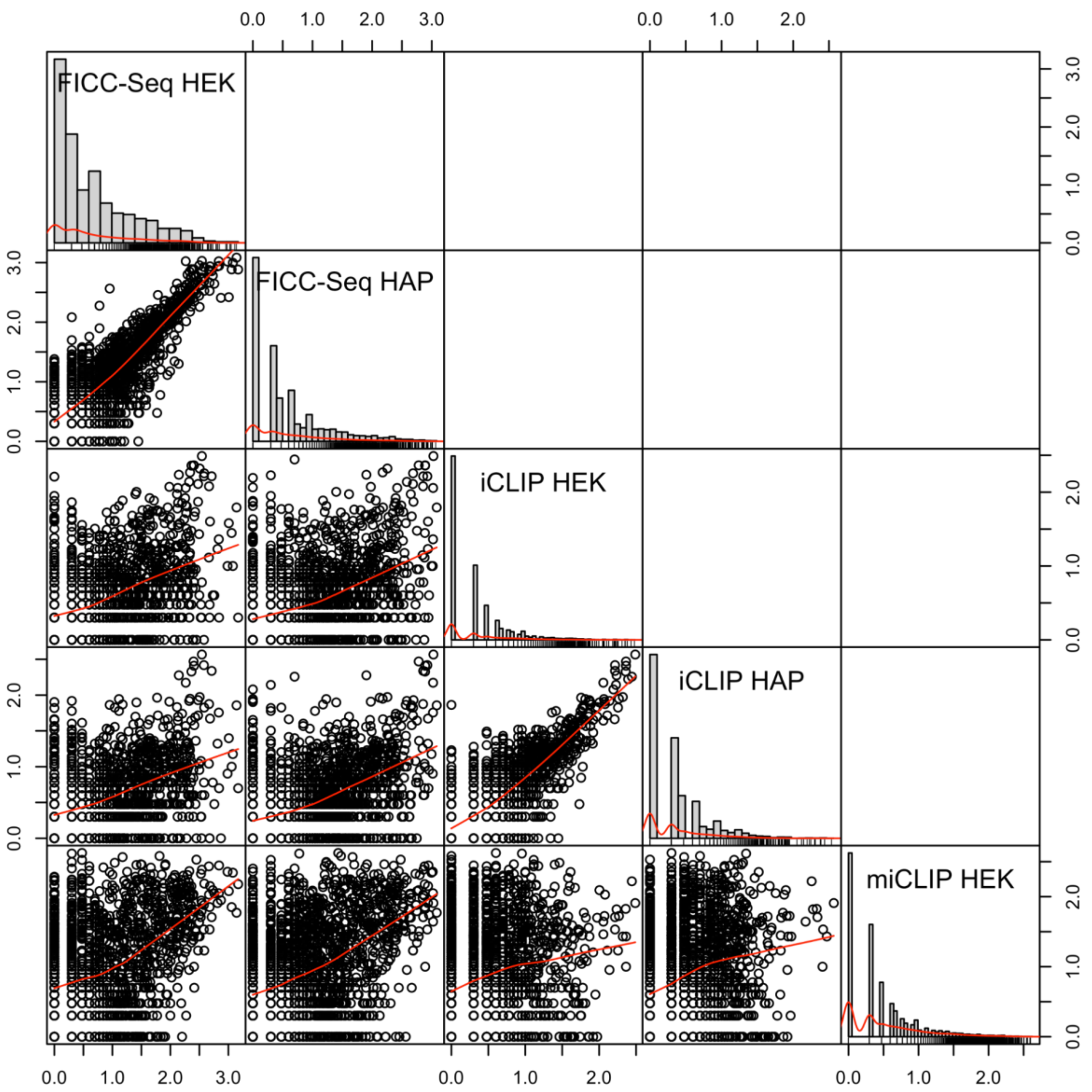

Figure S7. Scatterplots corresponding to Figure 5B showing inter-experimental comparison of crosslink peak position counts (FDR <0.05) for correlation estimates.

Supplementary Table 1. Normalised TRMT2A crosslink peak counts detected in FICC-Seq-HEK, FICC-Seq-HAP, iCLIP-HEK, iCLIP-HAP and miCLIP-HEK experiments; the table includes only sites with a minimum of 5 counts in at least 3 out of the 5 experiments.
